# Family origin and mortality: prospective Finnish cohort study

**DOI:** 10.1186/1471-2458-11-385

**Published:** 2011-05-25

**Authors:** Jan Saarela, Fjalar Finnäs

**Affiliations:** 1 Åbo Akademi University, P.O. Box 311, 65101 Vasa, Finland

## Abstract

**Background:**

Death rates are notably higher in eastern Finland than in western Finland, and life expectancy of Finnish speakers shorter than that of Swedish speakers. The mortality differences correspond to recent genetic mappings of the population and are prominent for causes of death that are known to be associated with genetic risk factors.

**Methods:**

Using intergenerational data, we studied the impact of parental birth area on all-cause mortality risks of middle-aged men in Finland 1985-2003, assuming that geographic family origin reflects genetic predisposition to complex disorders. Relative death risks at ages 30-49 years were estimated by parental birth region and ethnicity, according to Cox regressions standardised for own education, family type at childhood, and year of birth.

**Results:**

The death risk of Finnish speakers born in eastern Finland was 1.13 (95% confidence interval 1.01 to 1.26) that of Finnish speakers born in western Finland, whereas that of Swedish speakers was only 0.60 (0.52 to 0.71). In Finnish speakers, the effects of own birth area and area of residence disappeared when parental birth area was accounted for. The death risk of persons with at least one parent born in eastern Finland was 1.23 (1.09 to 1.39) that of people with both parents born in western Finland.

**Conclusions:**

Parental birth area is the driving force behind the regional mortality difference in Finland. The findings highlight and give further support for the potentially important role of genetic risk factors in mortality. Close monitoring of persons' geographic and ethnic ancestry may promote public health and avoid many early deaths.

## Background

Two of the most distinctive features of overall mortality in Finland are that death rates increase in the southwest to northeast direction and that life expectancy of Finnish speakers is shorter than that of Swedish speakers [1]. Both these aspects conform to recent genetic mappings [2,3]. There are differences in the genetic architecture that increase in the southwest to northeast direction, and Swedish speakers in Finland have been found genetically closer to Swedes than to Finnish speakers. The variation owes to the country's settlement history. Finns are unique on the genetic map of Europe, because they were at one time a small and geographically isolated population.

Being born in a high-mortality area in Finland is associated with a 10-30 per cent higher risk of dying from ischemic heart disease at high ages, as compared with being born in a low-mortality area [4]. A similar geographical pattern dominated by people's birth region has been observed for deaths from other common causes and in various age groups [5-7]. Hence, hereditary components might lie behind many deaths from cardiovascular diseases as well as external causes such as suicides and alcohol-related deaths. Many diseases, such as coronary heart disease, psychiatric disorders and alcoholism, are nowadays known to be associated with genetic susceptibility [8-10].

Taken together, these findings illustrate that, in the particular case of Finland, large-scale register data have the potential of shedding light on the question of how human genetic diversity manifests in mortality risks at the population level, even if they lack explicit genetic information and biomarkers. The country's settlement history consequently provides a rationale for interpreting the regional mortality differences as reflecting geographic clustering of hereditary factors. No explicit link between the mortality variation and genetic differences has yet been established, however.

All previous studies on the effect of geographic ancestry on mortality risks in Finland have studied the influence of persons' own birthplace as compared to that of their current place of residence. That approach for reflecting geographic origin may be contaminated by the past decades' internal migration flows. The birthplace of a parent, on the other hand, is a substantially better indicator of geographic family origin, as it widens the time horizon and is not confounded by person-specific environmental or behavioural factors.

This paper constitutes the first large-scale study of the Finnish regional mortality differences with focus on the influence of parental birth area. The opportunity was provided by recently available intergenerational data from longitudinal population registers. Assuming that geographic family origin reflects hereditary factors that influence mortality, we expected to find an effect of parental birth area on death risks irrespective of where the study persons were born or living as adults. Hence, even if people were born and raised in a low-mortality area, they would have an elevated death risk if a *parent *was born in a high-mortality area.

## Methods

The data came from the longitudinal population registers of Statistics Finland and covered the period 1970-2003. The part used here consists of an eight per cent sample of all Finnish speakers, and an additional 50% sample of all Swedish speakers. The latter constitute a national minority of barely six per cent of the total population. Basic demographic and socioeconomic variables are available for every fifth year, based on the quinquennial population censuses. Deaths from all causes and emigration are known for each year. For all persons born after 1953, there is corresponding information for the parents.

The structure implies that individuals could be observed at most up to the year when they become 50 years. Since there are few deaths at childhood, adolescence, and young adulthood, we started observing individuals from the year in which they became 30 years. The analyses consequently concerned ages 30-49 years, the period 1985-2003, and people born 1953-1970. One-year death risks were estimated with Cox regressions using age as the duration variable.

**Figure 1:**
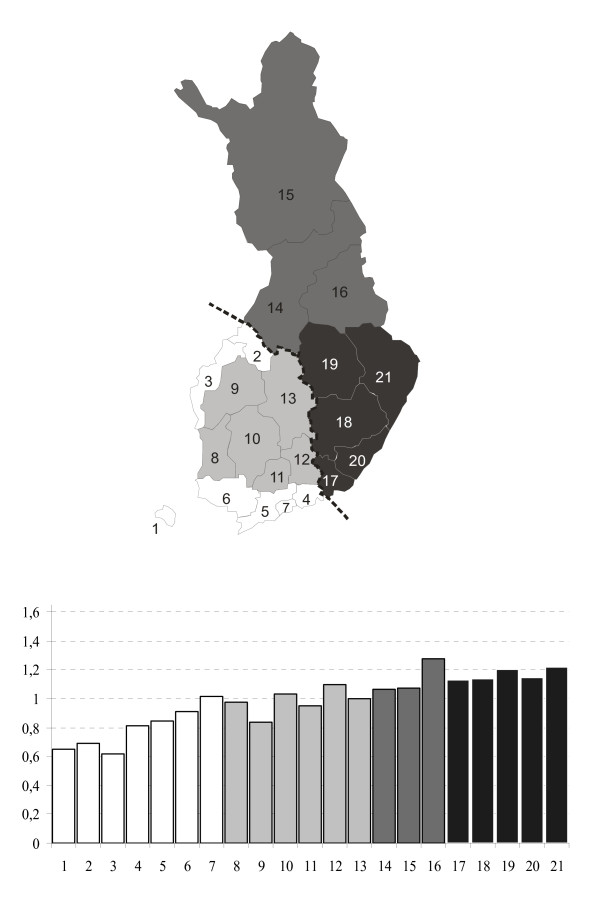
**Relative death rates by region in Finland, standardised for age and calendar year, men aged 35-49 years, 1986-2005 **. Total Finland is equal to one. The classification is according to the 20 administrative regions, plus one category (number 7) that separates the Helsinki metropolitan area. Authors' calculations based on [12].

Figure 1 illustrates the southwest to northeast increase in mortality rates standardised for age and calendar year, based on official aggregate statistics comprising the total male population by region for the period 1986-2005. The regions have been geographically numbered and coloured to facilitate comparisons. The thick broken line represents the most evident mortality difference, which roughly corresponds with the first national boundary between Sweden-Finland and Russia in 1323. A similar divide has been observed in terms of anthropology, folklore, and dialects [11].

In the data, there is unfortunately no information about the cause of death. However, from previous research we know that the regional mortality variation is dominated by mortality from cardiovascular diseases, alcohol-related causes and suicides [5], which also are the most common main causes of death in men aged 30-49 years (Table 1, which is based on [12]). Mortality in same-aged women is outside the scope of this paper, as it is modest and dominated by various non-vascular diseases that display no evident regional pattern.

**Table 1:** Annual number of deaths and distribution of main causes of death in ages 30-49 years in Finland, 1986-2005

	**Men **						**Women **					
		
	**#**	**%**	**%**	**%**	**%**	**%**	**#**	**%**	**%**	**%**	**%**	**%**
**Age group**	**Deaths**	**Cardio-vascular**	**Alcohol-related**	**Suicides**	**Other external**	**Other diseases**	**Deaths**	**Cardio-vascular**	**Alcohol-related**	**Suicides**	**Other external**	**Other diseases**
				
30-34	310	9.0	10.9	30.9	30.7	18.5	99	10.7	5.8	21.6	19.5	42.3
35-39	471	15.0	16.0	23.7	25.3	20.1	168	13.0	8.5	16.1	16.6	45.7
40-44	694	21.8	18.3	17.2	20.9	21.8	258	15.2	10.1	11.9	13.0	49.7
45-49	940	28.6	18.1	11.5	16.5	25.3	380	16.2	9.5	9.2	9.9	55.2

Annual number of deaths refers to the mean number per year for the period 1985 to 2005.

Main causes of death are according to Statistics Finland's 54-category short list of causes of death, which follows the ICD-10 classification. Cardiovascular causes refer to codes 27-30, Alcohol-related to code 41, Suicides to code 50, Other external to codes 42-49 and 51-54, and Other diseases to codes 1-26 and 31-40.

Authors' calculations based on [12].

To obtain enough statistical power for the analyses, a simplified regional categorisation was used. It distinguished two groups of Finnish speakers according to the east-west boundary mentioned above. For each region variable of interest - the person's area of residence (at age 30-34 years), the person's birth area, mother's birth area and father's birth area - the same categorisation was applied. A separate category was applied for the Swedish speakers, independent of their own birth area or parental birth area, because most of them originate in western Finland. As much as 97% of all Swedish speakers live in the regions that are white-coloured on the map, and only two per cent in other parts of the western area. The remaining one per cent was excluded from analysis.

We accounted for effects of some central personal characteristics in terms of each individual's birth year, educational level (primary, secondary, lower-degree tertiary, or higher-degree tertiary) and family type at childhood (one or two parents in the household at age 10-14 years). The latter was known because of the longitudinal structure of the data, meaning that all persons could be observed at the time when they were aged 10-14 years. All these variables are important mortality determinants in Finland [5-7]. There was information also about parental education and parental socioeconomic position. These variables were excluded from the models presented here, because they correlated strongly with the study persons' educational level, but had no impact on the estimates for the other variables.

## Results

The unweighted data comprised 61531 persons, whereof 47434 were Finnish speakers and 14097 were Swedish speakers (Table 2). The total number of deaths was 1464. The importance of internal migration on the population structure can be seen from the distribution of Finnish speakers on the region variables. Approximately 60% of the Finnish speakers were born in western Finland, but almost two thirds had at least one parent from eastern Finland. Even among those who were both born and residing in western Finland, the share with at least one parent born in eastern Finland was as high as 40% (see next section). Two thirds of the birth cohorts were living in western Finland at age 30 years.

**Table 2:** Persons' characteristics

**Variables**	**Number of persons**	**Percentage distribution**	**Number of deaths**
			
Area of residence at age 30-34 years (or ethnicity)			
Western Finland	31695	51.5	788
Eastern Finland	15739	25.6	469
Swedish speaker	14097	22.9	207
			
Birth area (or ethnicity)			
Western Finland	28016	45.5	688
Eastern Finland	19418	31.6	569
Swedish speaker	14097	22.9	207
			
Mother's birth area (or ethnicity)			
Western Finland	21930	35.6	521
Eastern Finland	25504	41.4	736
Swedish speaker	14097	22.9	207
			
Father's birth area (or ethnicity)			
Western Finland	22233	36.1	516
Eastern Finland	25201	41.0	741
Swedish speaker	14097	22.9	207
			
Parents' birth area (or ethnicity)			
Both from western Finland	16895	27.5	375
One from eastern Finland	10373	16.9	287
Both from eastern Finland	20166	32.8	595
Swedish speaker	14097	22.9	207
			
Educational level			
Primary	12668	20.6	556
Secondary	29509	48.0	715
Lower-degree tertiary	12665	20.6	144
Higher-degree tertiary	6689	10.9	49
			
Family type at age 10-14 years			
Lived with both parents	55963	91.0	1319
Lived with one parent	5568	9.0	145
			
Birth year			
1953-1955	10323	16.8	486
1956-1960	17369	28.2	555
1961-1965	17457	28.4	300
1966-1970	16382	26.6	123
			
Total	61531	100.0	1464

In a first model, we included persons' area of residence and the control variables. The results, which are summarised in Table 3 highlight some known facts. People residing in eastern Finland had 12% higher death risks (95% confidence interval 1.00 to 1.26) than those residing in western Finland, whereas the death risk of Swedish speakers was 40% lower. In these ages, the ethnic-group mortality difference is the widest [6]. Also the control variables had substantial effects. Mortality risks decreased notably over educational levels and birth cohorts, whereas people raised in one-parent families had almost 40% higher death risks than those originating in two-parent households. The inclusion of these variables did not reduce the area or ethnic mortality difference, however.

**Table 3:** Mortality risk ratios (95% confidence intervals) in model with area of residence

Variables	Risk ratio		Wald(d.f.)	p-value
Area of residence at age 30-34 years (or ethnicity)				
Western Finland	Reference		58.60(2)	< 0.001
Eastern Finland	1.12	(1.00 to 1.26)		
Swedish speaker	0.60	(0.51 to 0.70)		
				
Educational level				
Primary	Reference		267.93(3)	< 0.001
Secondary	0.58	(0.52 to 0.65)		
Lower-degree tertiary	0.28	(0.23 to 0.34)		
Higher-degree tertiary	0.20	(0.15 to 0.27)		
				
Family type at age 10-14 years				
Lived with both parents	Reference		12.79(1)	< 0.001
Lived with one parent	1.37	(1.15 to 1.63)		
				
Birth year				
1953-1955	Reference		5.98(3)	0.11
1956-1960	0.98	(0.86 to 1.12)		
1961-1965	0.90	(0.77 to 1.06)		
1966-1970	0.76	(0.61 to 0.95)		

Number of persons is 61531. Number of deaths is 1464.

Wald statistic (degrees of freedom) and p-value are for the entire variable

We proceeded by focusing on the influence of each area variable (Table 4). The control variables were also included, but since the estimated effects were the same as reported above, they are not shown here. Replacing area of residence (model 1) with own birth area (model 2) or mother's birth area (model 3) slightly improved the Wald statistic and increased the area difference marginally. Father's birth area (model 4) had a stronger effect and improved the statistical fit relatively more. People with the father born in eastern Finland had 21% higher death risks (1.08 to 1.35) than those with the father born in western Finland.

When using either mother's birth area or father's birth area, the reference category is heterogeneous with respect to the study persons' geographic family origin, because the other parent may be born in eastern Finland. Simultaneously accounting for both parents' birth area (model 5) revealed that the offspring death risk was 23% higher (1.09 to 1.39) if at least one parent was born in eastern Finland, as compared with people whose both parents were born in western Finland. Having both parents from eastern Finland was associated with only a slightly higher mortality risk as compared with having only one parent from eastern Finland, however (model 6).

**Table 4:** Mortality risk ratios (95% confidence intervals) in models with alternative area variables

Model	Variable	Risk ratio		Wald(d.f.)	p-value
Model 1	Area of residence at age 30-34 years (or ethnicity)				
	Western Finland	Reference		58.60(2)	< 0.001
	Eastern Finland	1.12	(1.00 to 1.26)		
	Swedish speaker	0.60	(0.51 to 0.70)		

Model 2	Birth area (or ethnicity)				
	Western Finland	Reference		59.26(2)	< 0.001
	Eastern Finland	1.13	(1.01 to 1.26)		
	Swedish speaker	0.60	(0.52 to 0.71)		

Model 3	Mother's birth area (or ethnicity)				
	Western Finland	Reference		59.78(2)	< 0.001
	Eastern Finland	1.14	(1.02 to 1.27)		
	Swedish speaker	0.62	(0.52 to 0.72)		

Model 4	Father's birth area (or ethnicity)				
	Western Finland	Reference		65.78(2)	< 0.001
	Eastern Finland	1.21	(1.08 to 1.35)		
	Swedish speaker	0.64	(0.54 to 0.75)		

Model 5	Parents' birth area (or ethnicity)				
	Both from western Finland	Reference		66.87(2)	< 0.001
	One or both from eastern Finland	1.23	(1.09 to 1.39)		
	Swedish speaker	0.66	(0.56 to 0.78)		

Model 6	Parents' birth area (or ethnicity)				
	Both from western Finland	Reference		66.87(3)	< 0.001
	One from eastern Finland	1.23	(1.05 to 1.43)		
	Both from eastern Finland	1.24	(1.09 to 1.41)		
	Swedish speaker	0.66	(0.56 to 0.78)		

Number of persons is 61531. Number of deaths is 1464.

All models are adjusted for educational level, family type, and birth year.

Wald statistic (degrees of freedom) and p-value are for the entire variable.

The importance of parental birth area can also be illustrated by adding it into either model 1 or model 2. The effects of area of residence and own birth area then totally disappear (Table 5), suggesting a marginal role of area-specific current or early-life factors. Having a parent born in eastern Finland was still associated with more than 20% higher death risks as compared with having both parents born in western Finland.

**Table 5:** Relative mortality risks (95% confidence intervals) by area of residence and by birth area when including parental birth area

Model	Variables	Risk ratio		Wald(d.f.)	p-value
Model 1A	Area of residence at age 30-34 years				
	Western Finland	Reference		3.89(1)	0.05
	Eastern Finland	1.12	(1.00 to 1.26)		

Model 1B	Area of residence at age 30-34 years				
	Western Finland	Reference		0.12(1)	0.72
	Eastern Finland	1.02	(0.90 to 1.17)		
					
	Parents' birth area				
	Both from western Finland	Reference		8.06(1)	0.005
	One or both from eastern Finland	1.22	(1.06 to 1.40)		

Model 2A	Birth area				
	Western Finland	Reference		4.54(1)	0.03
	Eastern Finland	1.13	(1.01 to 1.26)		

Model 2B	Birth area				
	Western Finland	Reference		0.02(1)	0.89
	Eastern Finland	1.01	(0.88 to 1.16)		
					
	Parents' birth area				
	Both from western Finland	Reference		7.36(1)	0.007
	One or both from eastern Finland	1.23	(1.06 to 1.42)		

Number of persons is 47,434. Number of deaths is 1257. Swedish speakers are excluded here because these models contain more than one area variable.

All models are adjusted for educational level, family type, and birth year.

Wald statistic (degrees of freedom) and p-value are for the entire variable.

Another way to contrast the influence of geographic family origin against that of current and childhood environmental factors is to restrict the study group to people born and residing in western Finland. Doing so, we found that persons with at least one parent born in eastern Finland had 21% higher death risks (1.04 to 1.41) than those whose both parents were born in western Finland (Table 6). Hence, there was only a modest reduction in the effect of parental birth area as compared to that of model 5 in table 4.

**Table 6:** Mortality risk ratios (95% confidence intervals) by parental birth area for people born and residing in western Finland

Variable	Risk ratio		Wald(d.f.)	p-value
Parents' birth area (or ethnicity)				
Both from western Finland	Reference		45.44(2)	< 0.001
One or both from eastern Finland	1.21	(1.04 to 1.41)		
Swedish speaker	0.66	(0.56 to 0.78)		

Total number of persons (deaths) is 40966 (866), whereof 16150 (361) are people with both parents born in western Finland, 10719 (298) with one or both parents born in eastern Finland, and 14097 (207) who are Swedish speakers.

The model is adjusted for educational level, family type, and birth year.

Wald statistic (degrees of freedom) and p-value are for the entire variable.

## Discussion and conclusions

Our results correspond with known facts about mortality of middle-aged men in Finland. The death risk of Finnish speakers in western Finland is approximately ten per cent lower than that of Finnish speakers in eastern Finland, but 40% higher than that of Swedish speakers. These mortality differences resemble genetic mappings of the population, but no explicit link has yet been established.

As related to previous research based on population registers, which has documented mortality effects of people's own birth area and area of residence, we have here proceeded by using intergenerational data and focusing on parental birth area. We find that mortality differences by people's own birth area, which might proxy not only genetic factors but also early-life conditions, are fully explained by the parents' birth area. If at least one parent was born in eastern Finland, the death risk is over 20% higher as compared with if both parents were born in western Finland. A similar elevation is the case even if we restrict the study group to people born and residing in western Finland. Hence, geographic clustering of hereditary factors, as assumed reflected by parental birth region, seems to manifest in increased mortality risks.

A limitation of the study is that we have no information about specific causes of death. However, we do know that in the ages studied, the dominating causes of death may, at least to some extent, be genetically related. It also needs to be stressed that, since we cannot trace any other ancestors than the parents, parental birth area as used here is most likely an underestimate of the true effect of geographic ancestry on mortality. Since internal migration in modern time has been directed primarily towards the western parts of the country, the current population in this area is genetically mixed. Many persons with both parents born in western Finland are therefore likely heterogeneous with respect to the birth area of second or higher order generations. Identification of family origin several generations back in time would accentuate any hereditable component predisposing to complex disorders [13].

An alternative explanation to the sizeable effect of parental birth area on offspring mortality would be learned patterns of behaviour. In that case, parents' poor diet and inadequate coping with risks in life would be transferred to the next generation in a patriarchal manner. The role of such social inheritance, as opposed to that of genetic factors, cannot, in our opinion, be large. It would imply that, even if the children were born, raised and living in a low-mortality area, they have embraced parents' supposed unhealthy behaviours. It also postulates that there would be some latent factor associated with the eastern Finnish way of life that is highly detrimental for health, and independent of whether it is the mother or the father that carries it. In families where one parent is from eastern Finland and the other from western Finland, it must also dominate any socially protective effects from the parent born in the low-mortality area. Various mappings of the Finnish population's health behaviours have been undertaken during the past decades. An overall conclusion based on these surveys is that dietary practices and culturally determined lifestyle factors do not underlie the regional mortality differences [14]. On the contrary, there is ample evidence about substantial genetic diversity across regions, and of susceptibility to specific diseases according to people's geographic roots [4].

During the past decade, the annual number of deaths in middle-aged men in eastern Finland has been approximately 700. As compared to same-aged Finnish-speaking men in western Finland, the excess mortality is more than 100 deaths per year. As the geographic mortality divide is strongly present also at higher ages [7-9], the total effect is even larger above the middle ages. At ages 50-74 years, for instance, the annual number of deaths by cardiovascular diseases, alcohol-related causes, and suicides in eastern Finland is almost 2000. This corresponds to an excess mortality of more than 300 deaths as compared to western Finland. From a public health perspective, many early deaths may consequently be avoided if genetic predisposition is recognised via close monitoring of persons' family origin.

## Competing interests

The authors declare that they have no competing interests.

## Authors' contributions

Both authors contributed equally in conceiving and designing the study, analysing and interpreting the data, and preparing the manuscript.

## Pre-publication history

The pre-publication history for this paper can be accessed here:

http://www.biomedcentral.com/1471-2458/11/385/prepub
